# Human myeloid‐derived suppressor cell expansion during sepsis is revealed by unsupervised clustering of flow cytometric data

**DOI:** 10.1002/eji.202049141

**Published:** 2021-05-05

**Authors:** Marco De Zuani, Marcela Hortová‐Kohoutková, Ivana Andrejčinová, Veronika Tomášková, Vladimír Šrámek, Martin Helán, Jan Frič

**Affiliations:** ^1^ International Clinical Research Center St. Anne's University Hospital Brno Brno Czech Republic; ^2^ Department of Biology Faculty of Medicine Masaryk University Brno Czech Republic; ^3^ Department of Anaesthesiology and Intensive Care Faculty of Medicine Masaryk University Brno Czech Republic; ^4^ Department of Modern Immunotherapy Institute of Hematology and Blood Transfusion Prague Czech Republic

**Keywords:** Flow cytometry, Multidimensional clustering, Myeloid‐derived suppressor cells, Sepsis, Septic shock

## Abstract

Myeloid‐derived suppressor cells (MDSCs) are important regulators of immune processes during sepsis in mice. However, confirming these observations in humans has been challenging due to the lack of defined preparation protocols and phenotyping schemes for MDSC subsets. Thus, it remains unclear how MDSCs are involved in acute sepsis and whether they have a role in the long‐term complications seen in survivors. Here, we combined comprehensive flow cytometry phenotyping with unsupervised clustering using self‐organizing maps to identify the three recently defined human MDSC subsets in blood from severe sepsis patients, long‐term sepsis survivors, and age‐matched controls. We demonstrated the expansion of monocytic M‐MDSCs and polymorphonuclear PMN‐MDSCs, but not early‐stage (e)‐MDSCs during acute sepsis. High levels of PMN‐MDSCs were also present in long‐term survivors many months after discharge, suggesting a possible role in sepsis‐related complications. Altogether, by employing unsupervised clustering of flow cytometric data we have confirmed the likely involvement of human MDSC subsets in acute sepsis, and revealed their expansion in sepsis survivors at late time points. The application of this strategy in future studies and in the clinical/diagnostic context would enable rapid progress toward a full understanding of the roles of MDSC in sepsis and other inflammatory conditions.

## Graphical abstract

M‐MDSCs and PMN‐MDSCs are expanded during septic shock. PMN‐MDSCs frequency remains elevated months after recovery from sepsis. Unsupervised metaclustering confirms and complements the results obtained by conventional gating.

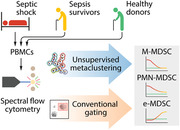

## Introduction

Myeloid‐derived suppressor cells (MDSCs) are a heterogeneous population of immature myeloid cells with strong immunosuppressive activity, especially on T cells and NK cells [1]. MDSCs are present at low frequencies in healthy donors (HD), but rapidly expand in pathological conditions including cancer, autoimmunity, and bacterial, fungal, and viral infections [1, 2, 3, 4, 5]. Although most of the studies proved that MDSCs play a pathologic role in these conditions by suppressing the protective immune response, few other reported that MDSC expansion might actually be beneficial, restraining potentially damaging inflammation [1, 6, 7, 8].

The MDSC population has classically been divided into two major subsets based on differences in morphology and phenotype: polymorphonuclear (PMN)‐MDSCs and monocytic (M)‐MDSCs. While murine MDSC phenotypes are widely accepted, studies of human MDSCs have used different markers to identify them and various methods of sample preparation [9, 10], leading to a lack of consensus. The most recent classification of human MDSCs identified three major subsets: monocytic myeloid‐derived suppressor cells (M‐MDSCs) (CD11b^+^, CD14^+^, HLA‐DR^–/lo^, CD15^–^), polymorphonuclear myeloid‐derived suppressor cells (PMN‐MDSCs) (CD15^+^, CD66b^+^, CD11b^+^, CD14^–^), and early‐stage (e)‐MDSCs (CD3/14/15/19/56^–^, HLA‐DR^–^, CD33^+^, CD11b^+^) comprising more immature myeloid progenitors [10]. How these subsets respond during the course of infection and inflammation, both in the acute phase of disease and during its resolution, is currently unknown.

Sepsis is a life‐threatening syndrome which is mainly caused by a dysregulated host response to pathogen infection, and affects approximately 50 million people worldwide every year [11]. Mouse models of acute sepsis show an accumulation of MDSCs, especially in the secondary lymphoid organs [12, 13]. However, the few studies that have investigated MDSCs’ role in acute human sepsis have used various sample preparation procedures and minimal flow cytometry panels to distinguish the different subsets [14], leaving the overall picture unclear. Some studies have indicated that MDSC frequency positively correlates with poor outcomes in acute sepsis patients, and may remain elevated for several weeks during recovery [15, 16]; however, a comprehensive analysis of MDSC subsets in sepsis patients and in long‐term survivors, who frequently exhibit chronic immunosuppression, is lacking.

Here, we used an advanced flow cytometric panel built on the most recent MDSC definition together with unsupervised clustering techniques to investigate the frequencies of the three main MDSCs subsets during acute sepsis, in sepsis survivors, and in HD.

## Results and discussion

**Figure 1:**
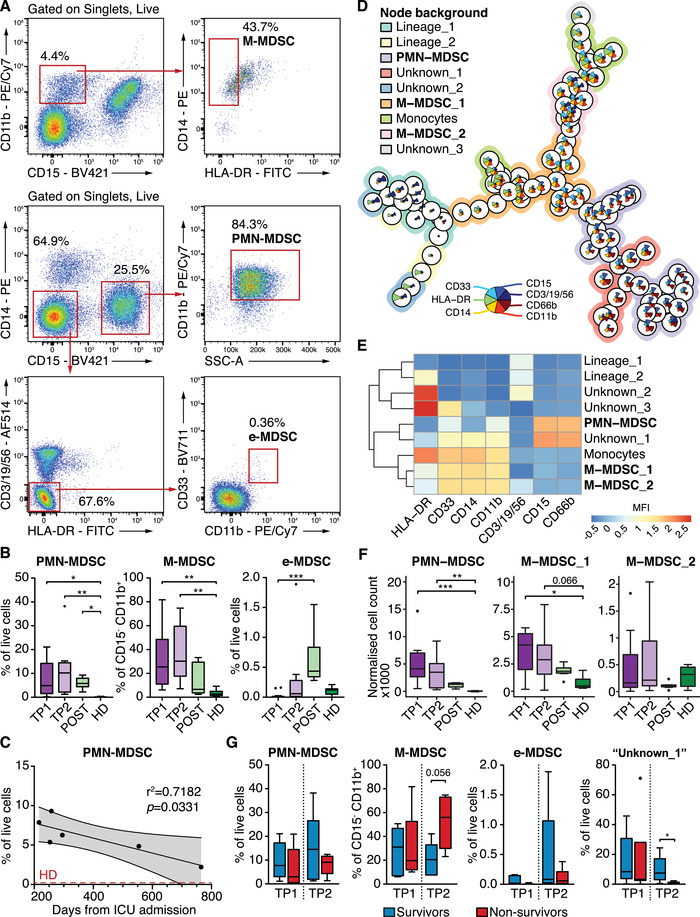
MDSCs are expanded during sepsis. (A) Representative gating strategy used to identify MDSCs. M‐MDSCs are defined as live, CD11b^+^, CD15^–^, CD14^+^, HLA‐DR^–^; PMN‐MDSCs are defined as live, CD15^+^, CD14^–^, CD11b^+^, SSC^hi^; e‐MDSCs are defined as CD3^–^, CD14^–^, CD15^–^, CD19^–^, CD56^–^, HLA‐DR^–^, CD33^+^, CD11b^+^. (B) Boxplots (Tukey) representing the frequency of M‐MDSCs, PMN‐MDSCs, and e‐MDSCs at different time points in acute sepsis patients (TP1, TP2) in sepsis survivors (POST), and in healthy donors (HD). Pairwise comparisons were performed using Dunn's test with multiple comparison correction. Asterisks indicate the level of significance: * *p ≤ *0.05, ** *p ≤* 0.005, *** *p *< 0.001. TP1, N = 12; TP2, N = 10; POST, N = 6; HD, N = 7. (C) Linear regression of the frequency of PMN‐MDSCs against the number of days from initial ICU admission in survivors. Grey area indicates 95% confidence interval. The red dashed line indicates the average PMN‐MDSC frequency in healthy donors (HD). *p* value indicates the *F*‐test result testing the null hypothesis of a zero slope. (D) Minimum spanning tree representation of the self‐organizing map built on the complete dataset. The height of the star plots in each node represents the mean fluorescence intensity of each marker used for the clustering. The background color of each node identifies the nine metaclusters. (E) Heatmap showing the mean fluorescence intensity of the expression of the different markers in each metacluster. (F) Boxplots (Tukey) representing the normalized number of cells grouped in each metacluster at different time points and in healthy donors. Pairwise comparisons were performed using Dunn's test with Holm's correction for multiple comparisons. Asterisks indicate the level of significance: * *p *≤ 0.05, ** *p *≤ 0.005, *** *p *< 0.001. TP1, N = 12; TP2, N = 10; POST, N = 6; HD, N = 7. (G) Boxplots (Tukey) of MDSC subset frequencies at different time points (TP1, TP2) in patients that survived or succumbed to septic shock. Pairwise comparisons were performed using a Mann‐Whitney *U* test. TP1, survivors, N = 5; TP1, nonsurvivors, N = 7; TP2, survivors, N = 5; TP2, nonsurvivors, N = 5.

To investigate the changes in MDSC subset abundance during sepsis, we enrolled 12 patients affected by acute septic shock, 6 long‐term sepsis survivors, and 7 age‐matched HD (Table 1). We labelled peripheral blood mononuclear cells (PBMCs) to identify M‐MDSCs (CD11b^+^, CD14^+^, HLA‐DR^–/lo^, CD15^–^), PMN‐MDSCs (CD15^+^, CD66b^+^, CD11b^+^, CD14^–^), and e‐MDSCs (CD3/14/15/19/56^–^, HLA‐DR‐, CD33^+^, CD11b^+^) (Fig. 1A, Supporting information Fig. S1). When we gated these populations manually, we saw significantly higher frequencies of both M‐MDSC and PMN‐MDSC, but not e‐MDSCs, in septic patients at both time points (TP1, TP2), compared to HD (Fig. 1B). These results are in concert with other studies showing the accumulation of MDSCs in sepsis patients [6, 16, 17]. Interestingly, we found that PMN‐MDSCs, but not M‐ and e‐MDSCs, were also significantly more frequent in the blood of recovered patients than in HD, with their frequency showing a linear decrease with time (Fig. 1B and C, Supporting information Fig. S2). Typically, recovered patients exhibit persistent low‐grade inflammation and immunosuppression which results in poor functional independence, increased susceptibility to secondary infection and reduced survival [15, 18]. These results call for investigation of the potential role of MDSCs subsets in sepsis survivors.

**Table 1:** Cohort characteristics

Study cohort	N	12
Sex	Female	6 (50%)
	Male	6 (50%)
Age, mean (range)		64.75 (28‐77)
Comorbidities, mean		3.25
	Hypertension, n	7 (58.3%)
	Peripheral artery disease, n	4 (33.3%)
	Asthma, n	3 (25%)
	Coronary heart disease, n	4 (33.3%)
BMI [kg/m^2^], mean		27.24
Mortality	N	7 (58.3%)
Causative agent	Soft tissue infection, n	1 (8.3%)
	Urosepsis, n	5 (41.7%)
	Mediastinitis/empyema, n	2 (16.7%)
	Pneumonia, n	2 (16.7%)
	Unknown, n	2 (16.7%)
TP1	SOFA, mean	13.3
	CRP [mg/L], mean	240.41
	Leucocyte count [10^9^/L], mean	22.23
	Horowitz index, mean	204.84
	Noradrenalin dose [μg/kg/min], mean	0.382
TP2	SOFA, mean,	5.9
	CRP [mg/L], mean	81.88
	Leucocyte count [10^9^/L], mean	16.26
	Horowitz index, mean	295.65
	Noradrenalin dose [μg/kg/min], mean	0.01

Postsepsis cohort	N	6
Sex	Female	4 (66%)
	Male	2 (33%)
Age, mean (range)		69.50 (60‐76)
Comorbidities, mean		2.17
BMI [kg/m^2^], mean		28.78
Data at ICU admission	SOFA, mean	10.00
	CRP [mg/L], mean	386.78
	Leucocyte count [10^9^/L], mean	8.767
	Horowitz index, mean	218.04
	Noradrenalin dose [μg/kg/min], mean	0.09
Causative agent	Abdominal infection, n	2 (33.3%)
	Pneumonia, n	1 (16.7%)
	Mediastinitis/empyema, n	2 (33.3%)
	Combined, n	1 (16.7%)
Follow‐up	Leucocyte count [10^9^/L], mean	6.12

Control cohort	N	7
Sex	Female	4 (57%)
	Male	3 (43%)
Age, mean (range)		70.8 (63‐82)
Comorbidities, mean		2.4
BMI [kg/m^2^], mean		29.79
CRP [mg/L], mean		2.75

BMI, body mass index; CRP, C‐reactive protein; Horowitz index (PaO_2_/FiO_2_), lung function index defined as the ratio of partial pressure of oxygen in arterial blood (PaO_2_) to the inspiratory fraction of oxygen (FiO_2_); SOFA, sequential organ failure assessment score.

To further confirm the expansion of these MDSC subsets, we applied FlowSOM, an unsupervised multidimensional clustering of flow data based on self‐organizing maps and minimum spanning tree algorithms [19]. The analysis identified two M‐MDSC metaclusters and one PMN‐MDSC metacluster (Fig. 1D, Supporting information Fig. S3A), all consistently expressing MDSC markers (Fig. 1E, Supporting information Fig. S3B, Table S1). The event count in each metacluster confirmed the increased frequencies of both PMN‐MDSCs and M‐MDSCs during sepsis (Fig. 1F), as seen with manual gating. Contrarily to the results obtained through manual gating, the number of canonical CD14^+^, HLA‐DR^+^ monocytes proved to be similar between the four groups (Supporting information Fig. S3C and 3D). Furthermore, we detected an unconventional subset (CD66b^+^, CD15^+^, CD14^+^, HLA‐DR^–^, CD33^+^, CD11b^+^) which was significantly increased in the earliest time point of sepsis but returned to physiological levels in sepsis survivors (Fig. 1E “Unknown_1,” Supporting information Fig. S4A and 4B). Manual gating of this population confirmed its expansion at the earliest time point of sepsis, compared to HD (Supporting information Fig. S4C). A similar subset (CD14^+^, CD15^+^, CD11b^+^, CD33^+^, HLA‐DR^–^, Lin^–^) was described to be expanded in nonsmall lung cancer patients and to correlate with reduced overall and progression‐free survival [20]. Moreover, a novel population of PMN‐neutrophils expressing high levels of CD14 and characterized by a strong immunosuppressive phenotype was recently described in the spleen of tumor‐bearing mice [21].

Taken together, we demonstrate that both M‐MDSCs and PMN‐MDSCs but not e‐MDSCs are present at high levels in patients with early‐stage sepsis. Similarly, a CD14^+^ CD15^+^ CD66b^+^ HLA‐DR^–^ unconventional subset was expanded at the earliest time point of sepsis. Although other studies in sepsis patients have correlated the expansion of MDSCs with higher mortality, we did not find this to be the case at the time points measured here (Fig. 1G); thus, the dynamic changes in MDSC abundance throughout the clinical course of sepsis, and their possible association with severity of the condition, require further investigation.

## Concluding remarks

Based on our findings, we believe that MDSCs might play a dual role in the early and late response to septic shock. Initially, MDSCs might help to mitigate the systemic hyperinflammation observed in the early stages of sepsis, as seen in several in vivo murine models of sepsis, and in a cohort of patients affected by cystic fibrosis and bacterial infection [7, 8, 22]. However, subsequently, the high PMN‐MDSCs frequencies described in our postsepsis cohort might account for the long‐term morbidity and high mortality observed in sepsis survivors [15, 16]. Although this cohort did not include subjects previously enrolled in the earlier time points, our data suggest that targeting MDSCs in long‐term sepsis survivors might improve overall survival and quality of life. For example, in cancer patients, treatment with all‐trans‐retinoic acid promoted the differentiation of MDSCs, reduced their numbers, and improved T‐cell responses [23, 24]. However, further investigations on sepsis survivors are encouraged, for example, to assess the immunosuppressive functions of the described MDSC subsets.

Finally, we demonstrate that unsupervised metaclustering algorithms are able to quickly identify the expansion of target immune subsets in clinical settings, thus, showing high potential for future use in operator‐free screenings and diagnostics. Moreover, these algorithms proved to be useful for the identification of unknown or noncanonical cell subsets, even when analyzing datasets obtained with limited flow cytometry panels.

## Methods

### Study participants

Twelve adult patients admitted to the intensive care unit (ICU) of the St. Anne's University Hospital in Brno with early septic shock were prospectively enrolled into the “study cohort.” Patients with chronic immunosuppression or receiving antibiotic therapy for more than 2 days were not enrolled. Blood and plasma samples were obtained at two time points: within 12 h (TP1), and at 3 days (TP2) after ICU admission. Secondly, six patients who had been successfully treated for sepsis at our department were retrospectively enrolled at 6 to 26 months from their initial ICU admission into the “postsepsis cohort.” Finally, seven healthy individuals of comparable age and health status were recruited into the “control cohort.” Patients with acute infection in the last 28 days or chronic immunosuppression were not enrolled in the study. Cohort details are summarized in Table 1.

Written informed consent was obtained from all enrolled patients. All procedures and protocols were approved by the institutional ethics committee (4G/2018).

### Sample collection and preparation

All samples were processed within 2 h from collection. Blood was collected in BD Vacutainer^®^ Tubes containing Sodium Heparin. PBMCs were isolated from 5 mL of heparinized blood by gradient centrifugation over Lymphoprep™ (1.077 g/mL, Alere Technologies AS, Norway) following manufacturer's recommendations. Cells were washed with FACS buffer (PBS with 0.5% FBS and 2 mM EDTA) and immediately labelled for flow cytometric analyses.

### Flow cytometry

Flow cytometry analyses were performed according to the guidelines published in Cossarizza, Chang [25]. A total of 10^6^ PBMCs were labelled in FC buffer for 30 min on ice with the following antibodies (dilutions, clones and vendors in brackets): anti‐CD3‐biotin (1:100, UCHT1, eBioscience), anti‐CD19‐biotin (1:100, HIB19, eBioscience), anti‐CD56‐biotin (1:50, CMSSB, eBioscience), anti‐CD11b‐PE/Cy7 (1:100, ICRF44, eBioscience), anti‐CD14‐PE (1:100, 61D3, eBioscience), anti‐CD15‐BV421 (1:100, W6D3, Sony Biotechnologies, San Jose, CA), anti‐CD66b‐PB (1:100, G10F5, Sony Biotechnologies), anti‐HLA‐DR‐FITC (1:50, L243, BioLegend), anti‐CD33‐BV711 (1:50, WM53, Sony Biotechnologies), and streptavidin‐AlexaFluor514 (5 μg/mL, Invitrogen). Propidium iodide was added immediately before sample acquisition to discriminate dead cells. Samples were acquired on a Sony SA3800 spectral analyser (Sony Biotechnologies).

### Data analysis

Data were imported and analyzed with FlowJo v10.7.1 (BD Life Sciences, Ashland, OR). For the unsupervised analysis, FCS files were imported into FlowJo, cleaned with FlowAI version 2.1, manually gated to exclude debris, doublets and dead cells, downsampled to match the event number across samples and exported to new FCS3 files [26]. Cleaned‐up files were processed with FlowSOM version 1.19.4 in the R version 4.0.2 environment [19]. The self‐organizing map was built on a 10 × 10 grid using a Manhattan distance function.

### Statistical analyses

Analyses were performed with Prism version 8.1 (GraphPad Software, San Diego, CA) or in R environment. Details of the statistical tests used are reported in each figure legend.

## Author contributions

MDZ conceived the project, designed and performed experiments, analyzed data, and drafted the manuscript; MHK and IA participated in sample processing and data collection; VT and MH recruited the patients and collected blood samples; MH and VŠ prepared the study protocol and the inclusion/exclusion criteria for patient enrolment; JF conceived and supervised the project, secured funding, and oversaw the writing of the manuscript. All authors read and approved the final manuscript.

## Conflict of interest

The authors declare no conflict of interest.

### Peer review

The peer review history for this article is available at https://publons.com/publon/10.1002/eji.202049141.

**Abbreviations**
e‐MDSC: early‐stage myeloid‐derived suppressor cells
HD: healthy donors
ICU: intensive care unit
MDSCs: myeloid‐derived suppressor cells
M‐MDSC: monocytic myeloid‐derived suppressor cells
PMN‐MDSC: polymorphonuclear myeloid‐derived suppressor cells

## Supporting information



Supporting Information

## Data Availability

Original data are available from the corresponding author upon reasonable request.

## References

[1] Veglia, F., Perego, M. and Gabrilovich, D., Myeloid‐derived suppressor cells coming of age. Nat. Immunol. 2018. 19: 108–119. 29348500 10.1038/s41590-017-0022-x PMC5854158

[2] Wu, H., Zhen, Y., Ma, Z., Li, H., Yu, J., Xu, Z. G., Wang, X. Y. et al., Arginase‐1‐dependent promotion of TH17 differentiation and disease progression by MDSCs in systemic lupus erythematosus. Sci. Transl. Med. 2016. 8: 331ra40. 10.1126/scitranslmed.aae0482 PMC4895207 27009269

[3] El Daker, S., Sacchi, A., Tempestilli, M., Carducci, C., Goletti, D., Vanini, V., Colizzi, V. et al., Granulocytic myeloid derived suppressor cells expansion during active pulmonary tuberculosis is associated with high nitric oxide plasma level. PLoS One. 2015. 10: e0123772. 25879532 10.1371/journal.pone.0123772 PMC4400140

[4] Rieber, N., Singh, A., Oz, H., Carevic, M., Bouzani, M., Amich, J., Ost, M. et al., Pathogenic fungi regulate immunity by inducing neutrophilic myeloid‐derived suppressor cells. Cell Host Microbe. 2015. 17: 507–514. 25771792 10.1016/j.chom.2015.02.007 PMC4400268

[5] Huang, A., Zhang, B., Yan, W., Wang, B., Wei, H., Zhang, F., Wu, L. et al., Myeloid‐derived suppressor cells regulate immune response in patients with chronic hepatitis B virus infection through PD‐1‐induced IL‐10. J. Immunol. 2014. 193: 5461–5469. 25344470 10.4049/jimmunol.1400849

[6] Uhel, F., Azzaoui, I., Gregoire, M., Pangault, C., Dulong, J., Tadie, J. M., Gacouin, A. et al., Early expansion of circulating granulocytic myeloid‐derived suppressor cells predicts development of nosocomial infections in patients with sepsis. Am. J. Respir. Crit. Care Med. 2017. 196: 315–327. 28146645 10.1164/rccm.201606-1143OC

[7] Sander, L. E., Sackett, S. D., Dierssen, U., Beraza, N., Linke, R. P., Muller, M., Blander, J. M. et al., Hepatic acute‐phase proteins control innate immune responses during infection by promoting myeloid‐derived suppressor cell function. J. Exp. Med. 2010. 207: 1453–1464. 20530204 10.1084/jem.20091474 PMC2901069

[8] Rieber, N., Brand, A., Hector, A., Graepler‐Mainka, U., Ost, M., Schafer, I., Wecker, I. et al., Flagellin induces myeloid‐derived suppressor cells: implications for *Pseudomonas aeruginosa* infection in cystic fibrosis lung disease. J. Immunol. 2013. 190: 1276–1284. 23277486 10.4049/jimmunol.1202144

[9] Damuzzo, V., Pinton, L., Desantis, G., Solito, S., Marigo, I., Bronte, V. and Mandruzzato, S., Complexity and challenges in defining myeloid‐derived suppressor cells. Cytometry B Clin. Cytom. 2015. 88: 77–91. 25504825 10.1002/cyto.b.21206 PMC4405078

[10] Bronte, V., Brandau, S., Chen, S. H., Colombo, M. P., Frey, A. B., Greten, T. F., Mandruzzato, S. et al., Recommendations for myeloid‐derived suppressor cell nomenclature and characterization standards. Nat. Commun. 2016. 7: 12150. 27381735 10.1038/ncomms12150 PMC4935811

[11] Rudd, K. E., Johnson, S. C., Agesa, K. M., Shackelford, K. A., Tsoi, D., Kievlan, D. R., Colombara, D. V. et al., Global, regional, and national sepsis incidence and mortality, 1990–2017: analysis for the Global Burden of Disease Study. Lancet. 2020. 395: 200–211. 31954465 10.1016/S0140-6736(19)32989-7 PMC6970225

[12] Delano, M. J., Scumpia, P. O., Weinstein, J. S., Coco, D., Nagaraj, S., Kelly‐Scumpia, K. M., O'Malley, K. A. et al., MyD88‐dependent expansion of an immature GR‐1(+)CD11b(+) population induces T cell suppression and Th2 polarization in sepsis. J. Exp. Med. 2007. 204: 1463–1474. 17548519 10.1084/jem.20062602 PMC2118626

[13] Makarenkova, V. P., Bansal, V., Matta, B. M., Perez, L. A. and Ochoa, J. B., CD11b+/Gr‐1+ myeloid suppressor cells cause T cell dysfunction after traumatic stress. J. Immunol. 2006. 176: 2085–2094. 16455964 10.4049/jimmunol.176.4.2085

[14] Schrijver, I. T., Theroude, C. and Roger, T., Myeloid‐derived suppressor cells in sepsis. Front. Immunol. 2019. 10: 327. 30873175 10.3389/fimmu.2019.00327 PMC6400980

[15] Mira, J. C., Gentile, L. F., Mathias, B. J., Efron, P. A., Brakenridge, S. C., Mohr, A. M., Moore, F. A. et al., Sepsis pathophysiology, chronic critical illness, and persistent inflammation‐immunosuppression and catabolism syndrome. Crit. Care Med. 2017. 45: 253–262. 27632674 10.1097/CCM.0000000000002074 PMC5243156

[16] Mathias, B., Delmas, A. L., Ozrazgat‐Baslanti, T., Vanzant, E. L., Szpila, B. E., Mohr, A. M., Moore, F. A. et al., Human myeloid‐derived suppressor cells are associated with chronic immune suppression after severe sepsis/septic shock. Ann. Surg. 2017. 265: 827–834. 27163951 10.1097/SLA.0000000000001783 PMC5102824

[17] Janols, H., Bergenfelz, C., Allaoui, R., Larsson, A. M., Ryden, L., Bjornsson, S., Janciauskiene, S. et al., A high frequency of MDSCs in sepsis patients, with the granulocytic subtype dominating in Gram‐positive cases. J. Leukoc. Biol. 2014. 96: 685–693. 24929004 10.1189/jlb.5HI0214-074R

[18] Gentile, L. F., Cuenca, A. G., Efron, P. A., Ang, D., Bihorac, A., McKinley, B. A., Moldawer, L. L. et al., Persistent inflammation and immunosuppression: a common syndrome and new horizon for surgical intensive care. J. Trauma Acute Care Surg. 2012. 72: 1491–1501. 22695412 10.1097/TA.0b013e318256e000 PMC3705923

[19] Van Gassen, S., Callebaut, B., Van Helden, M. J., Lambrecht, B. N., Demeester, P., Dhaene, T. and Saeys, Y., FlowSOM: using self‐organizing maps for visualization and interpretation of cytometry data. Cytometry A. 2015. 87: 636–645. 25573116 10.1002/cyto.a.22625

[20] Vetsika, E. K., Koinis, F., Gioulbasani, M., Aggouraki, D., Koutoulaki, A., Skalidaki, E., Mavroudis, D. et al., A circulating subpopulation of monocytic myeloid‐derived suppressor cells as an independent prognostic/predictive factor in untreated non‐small lung cancer patients. J. Immunol. Res. 2014. 2014: 659294. 25436215 10.1155/2014/659294 PMC4243712

[21] Veglia, F., Hashimoto, A., Dweep, H., Sanseviero, E., De Leo, A., Tcyganov, E., Kossenkov, A. et al., Analysis of classical neutrophils and polymorphonuclear myeloid‐derived suppressor cells in cancer patients and tumor‐bearing mice. J. Exp. Med. 2021. 218: e20201803. 33566112 10.1084/jem.20201803 PMC7879582

[22] Derive, M., Bouazza, Y., Alauzet, C. and Gibot, S., Myeloid‐derived suppressor cells control microbial sepsis. Intensive Care Med. 2012. 38: 1040–1049. 22552586 10.1007/s00134-012-2574-4

[23] Mirza, N., Fishman, M., Fricke, I., Dunn, M., Neuger, A. M., Frost, T. J., Lush, R. M. et al., All‐trans‐retinoic acid improves differentiation of myeloid cells and immune response in cancer patients. Cancer Res. 2006. 66: 9299–9307. 16982775 10.1158/0008-5472.CAN-06-1690 PMC1586106

[24] Iclozan, C., Antonia, S., Chiappori, A., Chen, D. T. and Gabrilovich, D., Therapeutic regulation of myeloid‐derived suppressor cells and immune response to cancer vaccine in patients with extensive stage small cell lung cancer. Cancer Immunol. Immunother. 2013. 62: 909–918. 23589106 10.1007/s00262-013-1396-8 PMC3662237

[25] Cossarizza, A., Chang, H. D., Radbruch, A., Acs, A., Adam, D., Adam‐Klages, S., Agace, W. W. et al., Guidelines for the use of flow cytometry and cell sorting in immunological studies (second edition). Eur. J. Immunol. 2019. 49: 1457–1973. 31633216 10.1002/eji.201970107 PMC7350392

[26] Monaco, G., Chen, H., Poidinger, M., Chen, J., de Magalhaes, J. P. and Larbi, A., Flowai: automatic and interactive anomaly discerning tools for flow cytometry data. Bioinformatics. 2016. 32: 2473–2480. 27153628 10.1093/bioinformatics/btw191

