# Supplementary material for: Human myeloid‐derived suppressor cell expansion during sepsis is revealed by unsupervised clustering of flow cytometric data
Source: Eur J Immunol. 2021 May 5;51(7):1785–91. doi: 10.1002/eji.202049141 (PMC8360154; doi:10.1002/eji.202049141)
Supplement: Supplementary file 1 — Supporting Information [file EJI-51-1785-s001.pdf]

Supplemental Figure 1

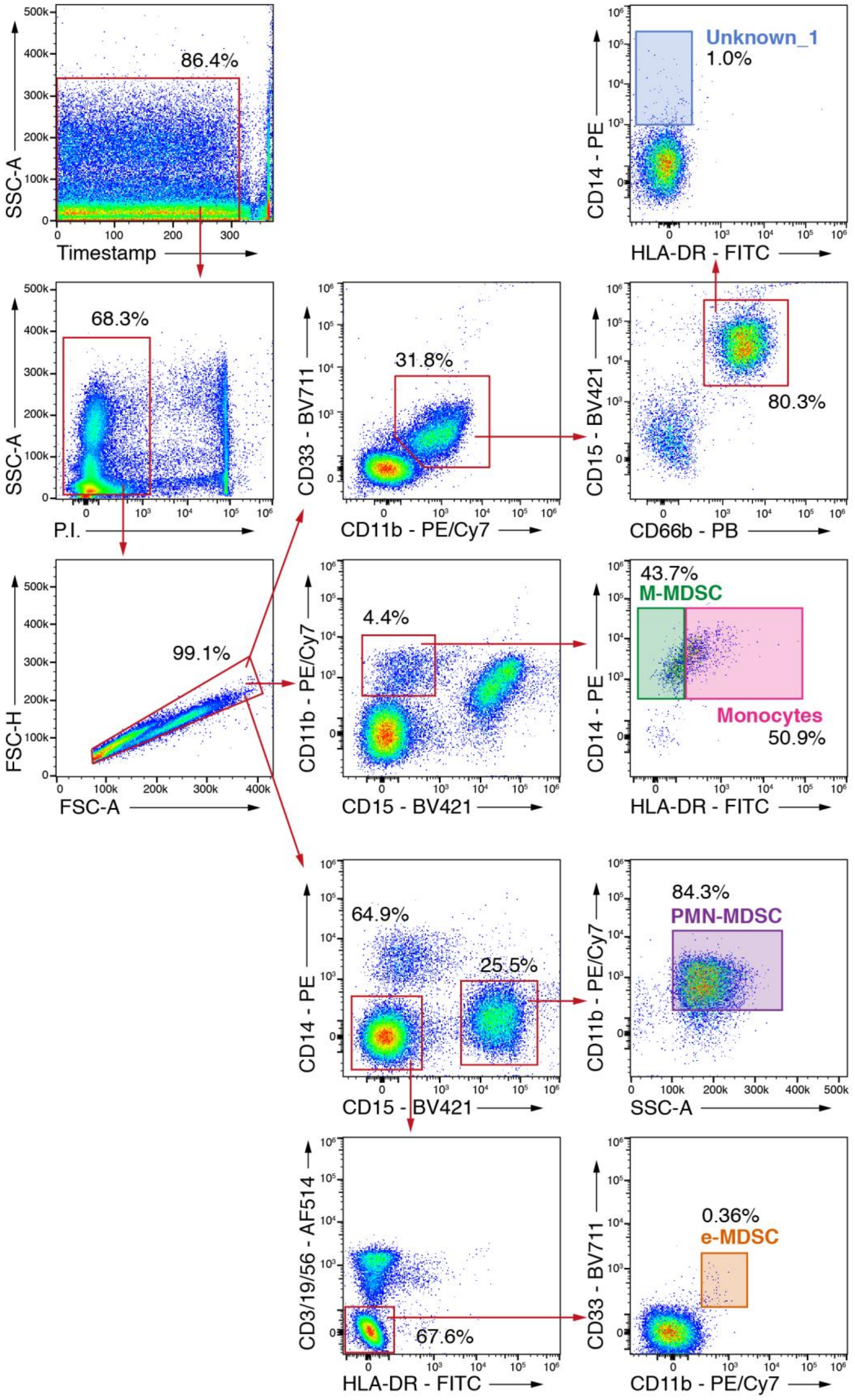

Supplemental Fig. 1: Compete gating strategy used to identify M-MDSCs, PMN-MDSCs, e-MDSCs, monocytes and the “Unknown_1” subset.

Supplemental Figure 2

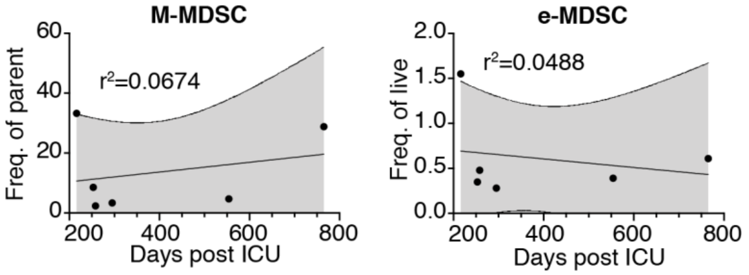

Supplemental Fig. 2: M-MDSC and e-MDSC counts in long-term sepsis survivors.

Supplemental Figure 3

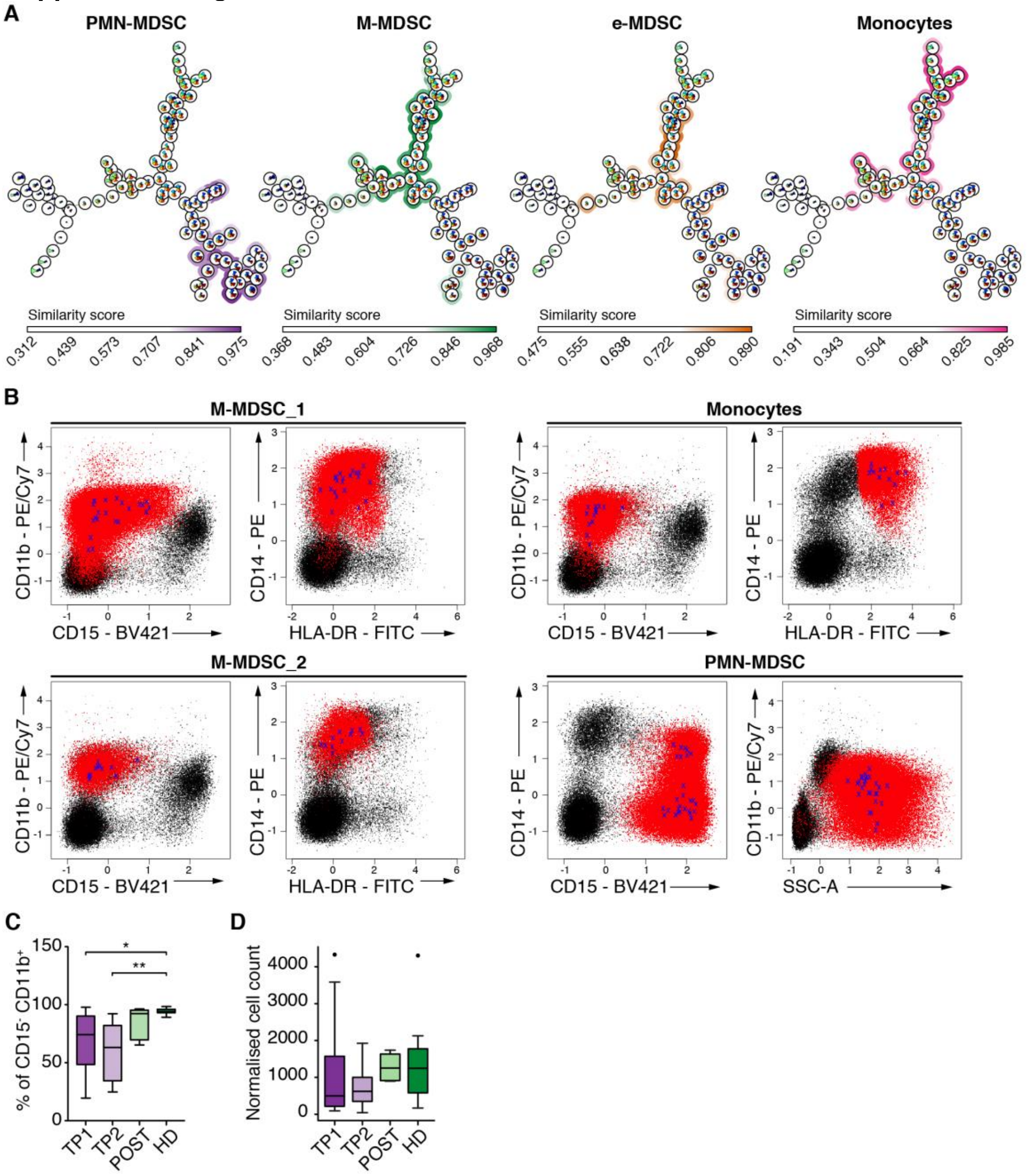

Supplemental Fig. 3: Unsupervised clustering confirms M-MDSC and PMN-MDSC expansion during sepsis. (A) The background intensity of the nodes in each minimum spanning tree plot corresponds to the degree of similarity with the training profile. Nodes with an "identity score" higher than 0.9 were used to identify the main metaclusters. PMN-MDSC were defined as CD15^{hi}, CD14^{lo}, CD11b^{hi}; M-MDSCs as CD15^{lo}, CD11b^{hi}, CD14^{hi}, HLA-DR^{lo}; e-MDSCs as CD3/19/56^{lo}, CD14^{lo}, CD15^{lo}, HLA-DR^{lo}, CD33^{hi}, CD11b^{hi}; monocytes as CD15^{lo}, CD33^{hi}, CD14^{hi}, HLA-DR^{hi}. **(B)** To confirm whether the identified metaclusters were phenotypically concordant with those of the manually-gated cells, the events in each metacluster were gated on the representative 2Dplots used for manually gating the MDSCs populations. **(C)** Boxplots (Tukey) representing the frequency of CD15^{-}, CD11b^{+}, CD14^{+}, HLA-DR^{+} monocytes resulting from manual gating. Pairwise comparisons were performed using Dunn's test with Holm's correction for multiple comparisons. Asterisks indicate the level of significance: * $p \leq 0.05$, ** $p \leq 0.005$. **(D)** Boxplots (Tukey) representing the normalized number of cells grouped in each metacluster at different time-points and in healthy donors. Pairwise comparisons were performed using Dunn's test with Holm's correction for multiple comparisons.

Supplemental Figure 4

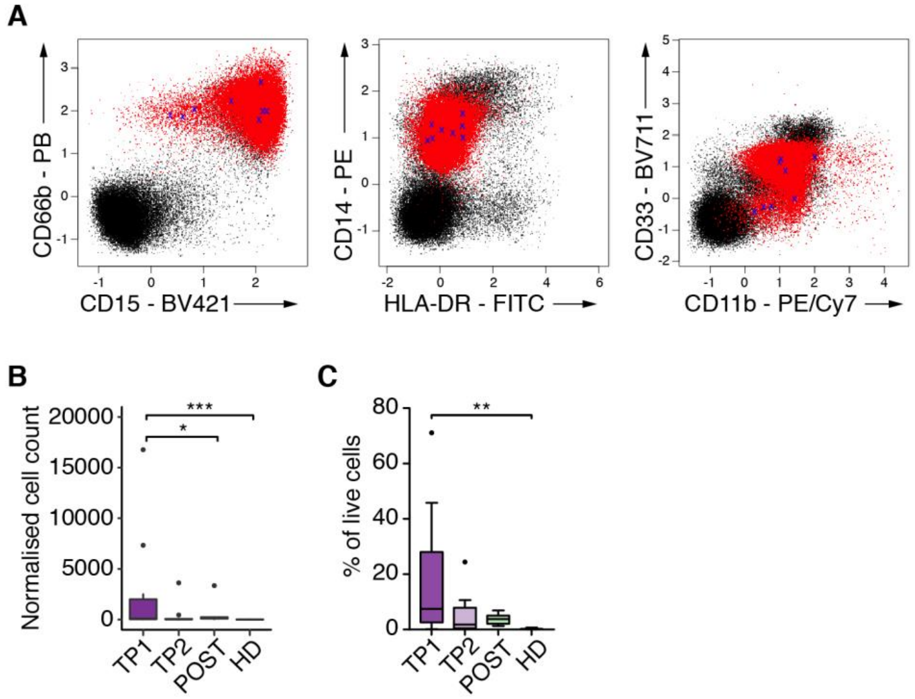

Supplemental Fig. 4: A CD14+ CD15+ CD66b+ HLA-DR- subset is expanded during sepsis. (A) Scatter plots showing the phenotype of the “Unknown_1” metacluster. **(B)** Boxplot (Tukey) representing the normalized number of cells grouped in the “Unknown_1” metacluster. Pairwise comparisons were performed using Dunn’s test with Holm’s correction for multiple comparisons. Asterisks indicate the level of significance: * $p \leq 0.05$, ** $p \leq 0.005$, *** $p \leq 0.001$. **(C)** Boxplot (Tukey) showing the fraction of “Unknown_1”-like cells among total live cells as identified by manual gating. Pairwise comparisons were performed using Dunn’s test with Holm’s correction for multiple comparisons. Asterisks indicate the level of significance: * $p \leq 0.05$, ** $p \leq 0.005$, *** $p \leq 0.001$.

Supplemental Table 1

	Manual gating	Meta-clustering
**M-MDSC**	$CD11b^+$, $CD15^-$, $CD14^+$, HLA-$DR^{-/lo}$	$CD15^{lo}$, $CD11b^{hi}$, $CD14^{hi}$, HLA-DR^{lo}
**PMN-MDSC**	$CD14^-$, $CD15^+$, $CD11b^+$, SSC^{hi}	$CD15^{hi}$, $CD11b^{hi}$, $CD66b^{hi}$, $CD14^{lo}$
**e-MDSC**	$Lin(CD3/19/56)^-$, $CD14^-$, $CD15^-$, HLA-DR^-,$CD33^+$, $CD11b^+$	$Lin(CD3/19/56)^{lo}$, $CD15^{lo}$, $CD33^{hi}$, HLA-DR^{lo},$CD14^{lo}$, $CD11b^{hi}$
**Monocytes**	$CD11b^+$, $CD15^-$, $CD14^+$, HLA-DR^+	$CD15^{lo}$, $CD33^{hi}$, $CD14^{hi}$, HLA-DR^{hi}
**Lineage**	-	$Lin(CD3/19/56)^{hi}$, $CD15^{lo}$, $CD33^{lo}$, HLA-DR^{lo}, $CD14^{lo}$, $CD11b^{lo}$, $CD66b^{lo}$

Supplemental Table 1: target phenotype of the cell subsets analysed either through manual gating or unsupervised meta-clustering.
